# Differential Regulation of TLR Signaling on the Induction of Antiviral Interferons in Human Intestinal Epithelial Cells Infected with Enterovirus 71

**DOI:** 10.1371/journal.pone.0152177

**Published:** 2016-03-23

**Authors:** Chunyang Wang, Lianfu Ji, Xinhui Yuan, Yu Jin, Carol J. Cardona, Zheng Xing

**Affiliations:** 1 Medical School, Jiangsu Provincial Key Laboratory of Medicine, and the State Key Laboratory of Pharmaceutical Technology, Nanjing University, Nanjing, China; 2 Nanjing Children’s Hospital, Nanjing Medical University, Nanjing, China; 3 Department of Veterinary Biomedical Sciences, College of Veterinary Medicine, University of Minnesota at Twin Cities, Saint Paul, Minnesota, United States of America; University of Tennessee Health Science Center, UNITED STATES

## Abstract

Enterovirus 71 (EV71) causes hand-foot-and-mouth disease, which can lead to fatal neurological complications in young children and infants. Few gastrointestinal symptoms are observed clinically, suggesting the presence of a unique immunity to EV71 in the gut. We reported a robust induction of interferons (IFNs) in human intestinal epithelial cells (HT-29), which was suppressed in other types such as RD and HeLa cells. The underlying mechanism for the apparent difference remains obscure. In this study we report that in EV71-infected HT-29 cells, TLR/TRIF signaling was essential to IFN induction; viral replication increased and the induction of IFN-α, -β, -ω, -κ, and -ε decreased markedly in TRIF-silenced HT-29 cells. Importantly, TRIF was degraded by viral 3C^pro^ in RD cells, but resisted cleavage, and IRF3 was activated and translocated into the nucleus in HT-29 cells. Taken together, our data suggest that IFNs were induced differentially in human HT-29 cells through an intact TLR/TRIF signaling, which differs from other cell types and may be implicated in viral pathogenesis in EV71 infection.

## Introduction

Enterovirus 71 (EV71) is a single-stranded RNA virus belonging to *Enterovirus* species A in the family Piconaviridae. The viral genome is approximately 7,500 nucleotides in length with a single open-reading frame that encodes a large polyprotein. During infection, this precursor polyprotein is proteolytically processed into four structural (VP1, VP2, VP3, and VP4) and seven nonstructural (2A, 2B, 2C, 3A, 3B, 3C, and 3D) proteins [1]. Mild cases of EV71 infection are usually characterized as childhood exanthema, also known as hand-foot-and-mouth disease. Acute EV71 infection can cause neurological syndrome, which may lead to permanent paralysis or even death [2, 3]. However, no effective vaccine or specific antiviral agents are currently available to prevent or treat EV71 infection [1, 4].

Virus infections tend to trigger production of interferons (IFNs) to block their spread. The IFN family mainly includes three classes of related cytokines: types I, II, and III IFNs, among which type I IFNs are mostly expressed in mammalian cells [5]. Type I IFNs mainly consist of IFN-α, β, ω, ε, and κ. By contrast, there is only one member of the type II family, IFN-γ, which possesses immunoregulatory as well as antiviral activities. IFN-γ is strongly produced by activated T cells or NK cells, but not by virus-infected cells [5]. IFN-λ1, λ2, and λ3, which belong to type III IFNs, are also induced by viral infection and have shown antiviral activities [6]. Among these cytokines, IFN-α and -β are the major effector cytokines in innate immunity against viral infections.

Studies have revealed that virus-associated components such as genomic DNA and RNA, or intermediate replicative double-stranded RNA (dsRNA), also known as pathogen-associated molecular patterns (PAMPs), are critical in the induction of IFNα/β through host pattern recognition receptors (PRRs), including Toll-like receptor (TLRs) [7] and RIG-I-like receptors (RLRs) [8, 9]. Viral single-stranded RNA and dsRNA are recognized in the endosome by TLR7/8 and TLR3, or in the cytoplasm by melanoma differentiation-associated protein 5 (MDA5)/retinoic acid-inducible gene 1 (RIG-I). TLRs on the endosomal membrane sense exogenous viral nucleic acids that have been endocytosed to the endosomes together with invading virions, whereas RLRs (MDA5 and RIG-I) in the cytoplasm recognize viral nucleic acids in the cytosol that subsequently relay the signal through an adaptor protein called mitochondrial anti-viral signaling (MAVS) on the mitochondrial membrane for signaling transduction [10]. Upon stimulation with poly (I:C), TLR3 is phosphorylated and primed to trigger a signaling cascade via an exclusive association with the TIR domain-containing adaptor protein inducing IFN-β, or TRIF (also known as TICAM-1) [11]. TRIF then associates with TRAF3 or TRAF6 through TRAF-binding motifs present in its N-terminus and interacts with downstream receptor-interacting protein 3 (RIP3) via the C-terminal domain [12–14]. Finally, a TLR3-TRIF-mediated signaling pathway leads to the activation of IRF3, IRF7, NF-κB, and AP1[15–17] and induction of antiviral IFNs and inflammatory cytokines as previously described [18]. Studies have shown that viruses have developed a variety of strategies to interfere with or block cellular signaling at various points to evade or subvert host immune responses.

As the entry site for EV71 infection, the intestinal tract is important for viral replication and spread during EV71 infection. Host responses in gastrointestinal epithelium may have significant impacts on EV71 pathogenesis, although this remains to be further elucidated. In our previous report, we found that EV71 infection induced robust IFN-β production in human colorectal adenocarcinoma HT-29 cells, but this did not occur in RD or HeLa cells [18–20]. In this study, we further examined the difference in IFN responses between these cell types and sought to elucidate the underlying mechanism by which the difference occurs during EV71 infection. We demonstrated that EV71 infection induced a robust production of type I IFNs independent of RIG-I or MDA-5 activation and signaling in HT-29 cells. Instead, induction of IFN was triggered through a TLR3/TRIF-dependent pathway in human intestinal epithelial cells, resulting in the activation of IRF3 and p-IRF3 translocation into the nucleus. Silencing of TRIF by a lentiviral vector expressing a specific shRNA in HT-29 cells led to remarkable downregulation of type I IFNs induced by EV71. While the TLR3-TRIF signaling was efficiently suppressed due to degradation of TRIF by viral proteins (3C^pro^ or 2A^pro^) in EV71-infected RD or HeLa cells, TRIF resisted proteolytic degradation in infected HT-29 cells. We concluded that in EV71-infected HT-29 cells, TRIF resisted viral proteolytic degradation, enabling subsequent activation of TRIF/IRF3 and functional signaling for antiviral IFN induction.

## Methods and Materials

### Cells and reagents

Human cervical carcinoma cell line (HeLa), human rhabdomyosarcoma cell line (RD), and human embryonic kidney 293T (HEK293T) cell line were cultured in Dulbecco’s modified Eagle’s medium (DMEM) with high glucose supplemented with 10% heat inactivated fetal bovine serum, 2 mM L-glutamine, non-essential amino acids, and sodium pyruvate (Invitrogen, Carlsbad, CA, USA). Human colorectal adenocarcinoma HT-29 cells, which are immortalized, were grown in RPMI-1640 medium (Invitrogen) supplemented with 10% fetal bovine serum (Sigma-Aldrich, St. Louis, MO), 0.1 mM non-essential amino acids (Gibco, Grand Island, NY, USA), 1 mM sodium pyruvate (Amresco, Solon, OH, USA), 1% antibiotic–antimycotic solution (Gibco), and 55 μM 2-mercaptoethanol (Amresco). The cells were cultured at 37°C in a humidified atmosphere with 5% CO_2_.

Anti-EV71 VP1 antibody was purchased from Millipore (Billerica, MA, USA). Antibodies specific for TRIF, MyD88, TRAF6, IRF7, TAK1, p-TAK1, TBK1, MDA5, MAVS, RIGI, IRF3, and p-IRF3 were purchased from Cell Signaling Technology (Beverly, MA, USA). Antibodies specific for human eIF4G were obtained from Millipore (Billerica, MA, USA), for GAPDH (MB001) from Bioworld Technology (Minneapolis, MN), and for β-actin (sc-130300) from Santa Cruz Biotechnology (Santa Cruz, CA, USA). HRP-conjugated anti-rabbit and anti-mouse IgG antibodies were obtained from Santa Cruz Biotechnology. The Super Signal ECL reagent kit was purchased from Thermo Fisher (Rockford, IL, USA). Fluorescein isothiocyanate (FITC)-conjugated donkey anti-mouse and rhodamine (TRITC)-conjugated goat anti-rabbit IgG antibodies were purchased from Cell Signaling Technology. Polyriboinosinic:polyribocytidylic acid [Poly(I:C)], a synthetic dsRNA compound, was purchased from Sigma-Aldrich (St. Louis, MO, USA).

### Virus and virus infection

The virus was propagated in Vero cells. Virus titers in the cells were determined by measuring 50% Tissue Cell Infectious Dose (TCID_50_), and the virus stocks were aliquoted and stored at -80°C. For the TCID_50_ assay, serially diluted viruses from 10^−2^ to 10^−9^ in DMEM were inoculated to Vero cells in 96-well plates. The cells were incubated for seven days at 37°C before titers were calculated by counting the wells with cytopathic effect (CPE) in infected Vero cells using the formula: logTCID_50_ = L−d (s−0.5), where L is the log of the lowest dilution, d is the difference between dilution steps, and s is the sum of the proportion of positive wells [21].

### Plasmids and transfection of viral protease genes

cDNA of 3C^pro^ was subcloned into an expression plasmid, pRK5-HA, for ectopic expression. RD and HT-29 cells were transfected with the plasmid for 36 hrs with Lipofectamine 2000 reagents (Invitrogen) for 3C^pro^ expression. Cell lysates were prepared and analyzed by western blot analysis.

### Cell Viability assay

Cell viability was determined using a methylthiazoletetrazolium (MTT) assay. Briefly, HT-29 cells were infected with lentiviruses expressing specific shRNA and cultured at 37°C for 72 hrs. MTT (0.5 mg/mL) was added into each well and the cells were incubated for another 4 hrs. Supernatants were removed and 200μL of dimethyl sulfoxide (DMSO) was added to each well. The cells were measured with a Microplate Reader (Unico, CA, USA) for absorbance at 570 nm. The survival rates of the cells were analyzed by calculating the ratio of optical density at 570 nm (OD_570_) of treated cells to that of untreated cells.

### Quantitative real-time RT-PCR and ELISA

Total RNA was isolated from uninfected and infected cells by using the RNeasy Miniprep Kit (Qiagen, Germany) following manufacturer’s instructions. The quantity and purity of total RNA were measured with a Nanodrop reader (Thermo Scientific, Waltham, MA, USA). 500 ng of total RNA was reverse transcribed using reverse transcriptase M-MLV (RNase H-) (Invitrogen). Real-time PCR was performed using the SYBR^®^ qPCR Mix (TOYOBO, Osaka, Japan). The 2^−ΔΔCt^ method [22] was used to quantify and normalize the relative quantification data. Data were calculated as fold change (2^−ΔΔCt^), which was the copy numbers of corresponding gene transcripts normalized to an internal control, glyceraldehyde-3-phosphate dehydrogenase (GAPDH). Assays were implemented in duplicate and repeated three times for each sample, and the mean values and standard deviations were calculated. The protein levels of IFN-α/β in culture supernatants were determined with ELISA kits (Bio-Rad Laboratories, Hercules, CA, USA) as previously described [23].

### Preparation of the cytoplasmic and nuclear extracts

RD cells were infected with EV71 for 24 hrs or stimulated with 50 μg poly (I:C) /ml in the cultural medium for 4 hrs at 37°C. Unstimulated and uninfected cells were used as controls. Cell lysates, cytoplasmic, and nuclear extracts were prepared using the Nuclear and Cytoplasmic Protein Extraction Kit according to the manufacturer’s instructions (P0027, Beyotime Institute of Biotechnology, Haimen, Jiangsu, China). The lysates or nuclear extracts were quantified using a BCA Protein Assay kit (BPA, Beyotime Institute of Biotechnology).

### Western blot analysis

Cell lysates from infected cells or non-infected cells were prepared and resolved by SDS-PAGE. Cellular proteins were transferred to PVDF membranes and probed with respective primary antibodies for western blot analysis. After overnight incubation at 4°C, the membranes were incubated with an HRP-conjugated secondary antibody for 1 hr at room temperature. Proteins of interest were visualized using Fluro Chem FC2 Imaging System (Alpha Innotech Corporation, Santa Clara, CA, USA) and developed manually.

### Immunofluorescence analysis

EV71-infected and non-infected HT-29 cells or RD cells grown on coverslips were rinsed twice with PBS at various time points post infection (p.i.), fixed with 4% paraformaldehyde for 30 mins, and permeabilized with 0.1% Triton X-100 for 10 mins, followed by three washes with PBS. The coverslips were blocked with 5% BSA (Sigma-Aldrich) in PBS for 30 mins at 37°C, then incubated with a primary antibody at a dilution of 1:100 at 4°C overnight. The cells were incubated with FITC-conjugated anti-mouse or TRITC-conjugated anti-rabbit IgG (H+L) antibodies. After three washes, the cells were incubated with 1 μg/ml DAPI in PBS for 5 mins. The coverslips were observed under an Olympus confocal fluorescence microscope.

### Generation of recombinant lentivirus and silence of TRIF in HT-29 cells

Lentiviruses were packaged by using Open Biosystems Trans-Lentiviral GIPZ Packaging System (Thermo Scientific) with lentiviral vector transfection in packaging cells following the manufacturer’s protocol. First, the Trans-Lentiviral Packaging mix and the transfer vectors, containing an shRNA targeting the gene of TRIF or a scramble shRNA as a control, were mixed and used to co-transfect TLK-293T packaging cells. After 48–72 hrs incubation, virus-containing supernatants were harvested and centrifuged to pellet cell debris. The resultant lentiviral particle titers were determined before the viruses were used to infect HT-29 cells at an m.o.i. of 10 with 8 μg/ml of polybrene (Sigma-Aldrich). After 24 hrs the medium was removed and replaced with fresh medium containing 0.2 μg/ml puromycin (Invitrogen) for another 5 days. During this period, the medium was replaced with fresh ones containing antibiotics when necessary, and expression of GFP in the cells was examined directly under a fluorescence microscope. Positive clones expressing GFP and resistant to puromycin were screened and recovered, and designated as HT-29-TRIF (TRIF shRNA) and HT-29-CTL (scramble shRNA control), respectively. The expression of TRIF in shRNA-silenced cells was detected by a realtime RT-PCR with primers specific for TRIF and western blot analysis. Finally, both HT-29-CTL and HT-29-TRIF cell lines were expanded and cultured for further experiments.

### Statistical analysis

Two-tailed Student’s *t*-test was used to evaluate the data. The data shown are the mean of three independent experiments ± SEM. The P value ≤ 0.05 level was considered statistically significant.

## Results

### Differential Induction of IFNs in cell cultures infected with EV71

**Fig 1:**
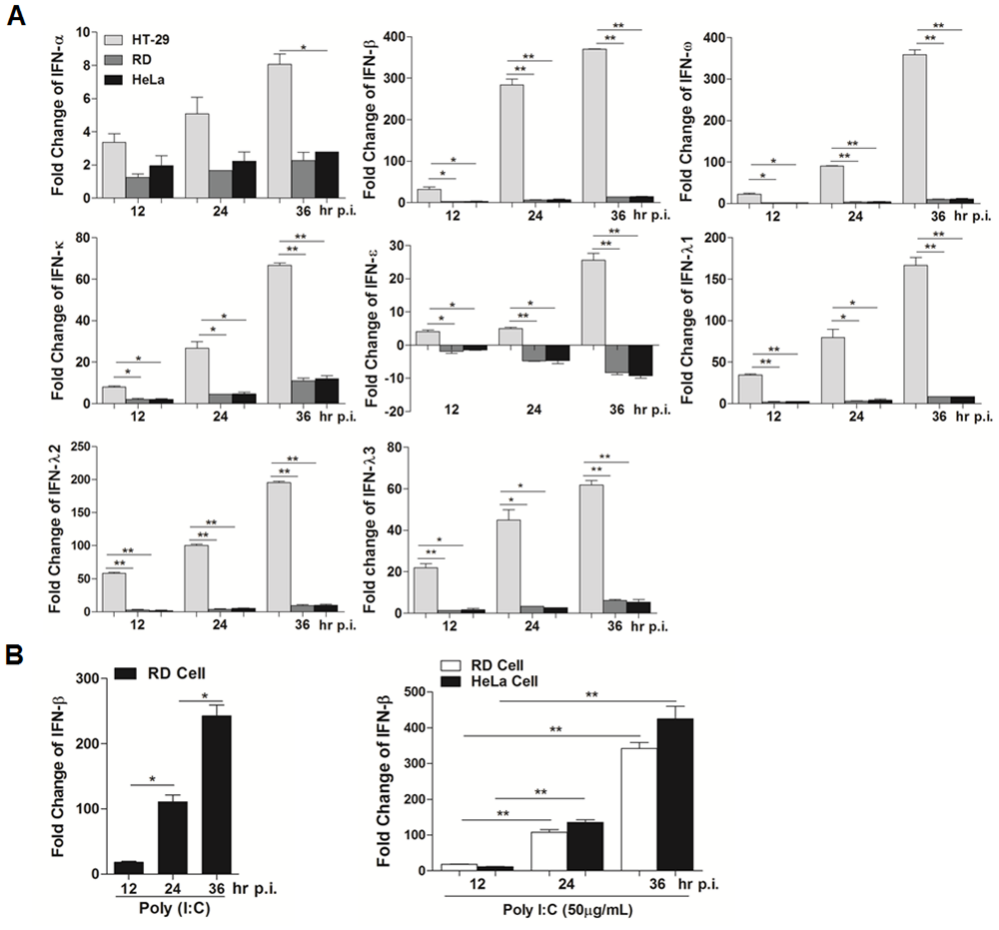
Differential induction of IFNs in cell cultures infected with EV71. **A**. HT-29, RD or HeLa cells were infected with EV71 at an m.o.i. of 2, and total RNA were prepared at indicated time points for reverse transcription. cDNA was used for realtime PCR with specific primers for IFN-α, IFN-β, IFN-ω, IFN-ε, IFN-κ, IFN-λ1, IFN-λ2, and IFN-λ3 for measuring transcript fold change of each gene in three cultures. **B**. RD cells and HeLa cells were stimulated with 50 μg poly (I:C) and incubated at 37°C. At indicated time points, total RNA was isolated and reverse transcribed to cDNA. The mRNA levels of IFN-β were measured by real time PCR with specific primers. The assays were repeated at least three times and statistical analyses were performed with a two-tailed Student *t* test (*p<0.05; **p<0.01).

Previous studies have shown that the induction of IFNs differed in human intestinal epithelial cells from other cell types infected with EV71, indicating that regulation of IFN induction could be differential among various cell types. To understand the difference, we infected HT-29 cells, RD cells, and HeLa cells with EV71 and comprehensively evaluated IFN responses in these cell types. The cells were infected with EV71 at a multiplicity of infection (m.o.i.) of 2. Total RNA was prepared at 12, 24, and 36 hrs p.i. and reverse transcribed to cDNA, which were used for realtime PCR with primers specific for IFN genes to measure copy numbers of each gene transcripts. As shown in Fig 1A, the expression pattern of Type I interferons, including IFN-α, -β, -ω, -κ, and -ε, and Type III interferons, IFN-λ1, -λ2, and λ3, was significantly different between HT-29 and RD/HeLa cells after EV71 infection. With the exception of IFN-α, which had a mild induction through 36 hrs p.i., all other type I and III IFNs were robustly induced up to dozen- (IFN-ε, IFN-κ, and IFN- λ3) or hundred- (IFN-β, IFN-γ, IFN- λ1, and IFN- λ3) fold increase at 36 hrs p.i. in infected HT-29 cells. In a striking contrast, no evident induction of either type I or type III IFNs was observed in RD or HeLa cells throughout the course of the infection. Our data demonstrate unambiguously that host responses are differentially regulated and antiviral IFN responses are effectively suppressed in RD and HeLa cells, but, on the contrary, were significantly induced in intestinal epithelial cells.

Both RD and HeLa cells were stimulated with poly (I:C) and total RNA were prepared from the cells at different time points after stimulation for realtime RT-PCR with primers specific for IFN-β. As shown in Fig 1B, transcripts of IFN-β were induced robustly in RD and, at earlier time, in HeLa cells, indicating that both cell lines produced IFN-b efficiently upon stimulation.

### Differential regulation of TRIF and downstream signaling in HT-29 and RD cells

**Fig 2:**
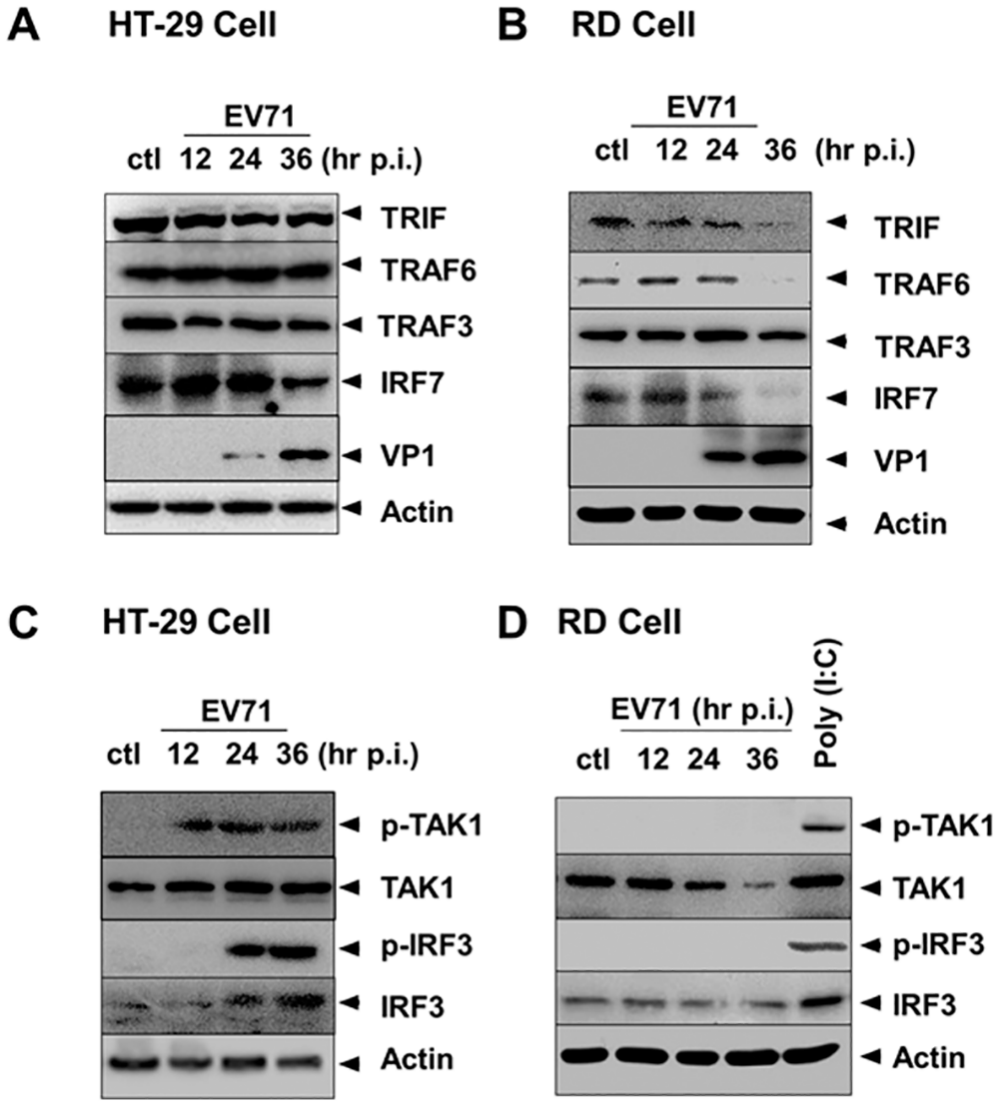
Differential regulation of TLR signaling in HT-29 and RD cells. HT-29 and RD cells were infected with EV71 at an m.o.i. of 2, and cell lysates were prepared at 12, 24, and 36 hrs p.i. for SDS-PAGE and western blot analysis with specific antibodies. **A** and **C**: HT-29 cells; **B** and **D**: RD cells. RD cells were also treated with poly (I:C) for 4 hrs and the lysates were used as a positive control for detecting the phosphorylated TAK1 and IRF3.

Virus infection induces IFN response through TLR and RLR signaling, which recognize viral RNA signals [15, 24]. To understand why the IFN induction in response to EV71 differs between HT-29 and other cells, we examined the expression and activation of signaling molecules important in these pathways. HT-29 and RD cells were infected with EV71 at an m.o.i. of 2, then total cell lysates were prepared at various time points p.i. and subjected to SDS-PAGE and western blot analysis. As shown in Fig 2A and 2B and S1 Fig, TRIF, the adaptor protein for MyD88-independent TLR signaling, remained largely unchanged in protein level in HT-29 cells, which relays signal to TRAF3 and activates TBK1 (Fitzgerald et al., 2003) and phosphorylates IRF3 (Fig 2C) for IFN induction [25]. However, in RD cells, TRIF decreased significantly as early as 12 hrs p.i. (Fig 2B and S1 Fig), which might compromise TLR/TRIF signaling. For MyD88-dependent TLR signaling, the level of IRF7 remained unchanged throughout 24 hrs of infection in HT-29 cells, which might be affected negatively in RD cells as well, since IRF7 decreased in quantity early after infection (Fig 2A and 2B). As a result, no phosphorylated IRF3 could be detected in infected RD cells (Fig 2D). At this stage we do not know which pathway, MyD88-independent TLR3 or MyD88-dependent TLR7/8, is more important in IFN signaling during EV71 infection. But in either case, TLR signaling would be compromised in RD cells, while not affected in HT-29 cells.

TRAF3, an upstream activator of TBK1, remained constant in quantity and was not degraded in either HT-29 or RD cells (Fig 2A and 2B), but TRAF6 seemed to disappear at 36 hrs p.i. only in RD cells. It is well accepted that TRIF also associates with TRAF6, an upstream activator of transforming growth factor-β-activated kinase 1 (TAK1), while TAK1 will activate NFκB signaling [26] leading to induction of both IFN and inflammatory cytokines. Remarkably, TAK1 was phosphorylated strongly in HT-29 cells, but no phosphorylated TAK1 was detected throughout the infection in RD cells (Fig 2C and 2D), indicating that unlike in RD cells, both IRF and NFκB signaling pathways from TLR remained intact and activated in EV71-infected HT-29 cells (Wang et al., 2015). We noted that RD cells are capable of IFN induction (Fig 1B) and TAK1 phosphorylation (Fig 2D) after stimulation with 50 μg poly (I:C)/ml in the medium for 4 hrs.

**Fig 3:**
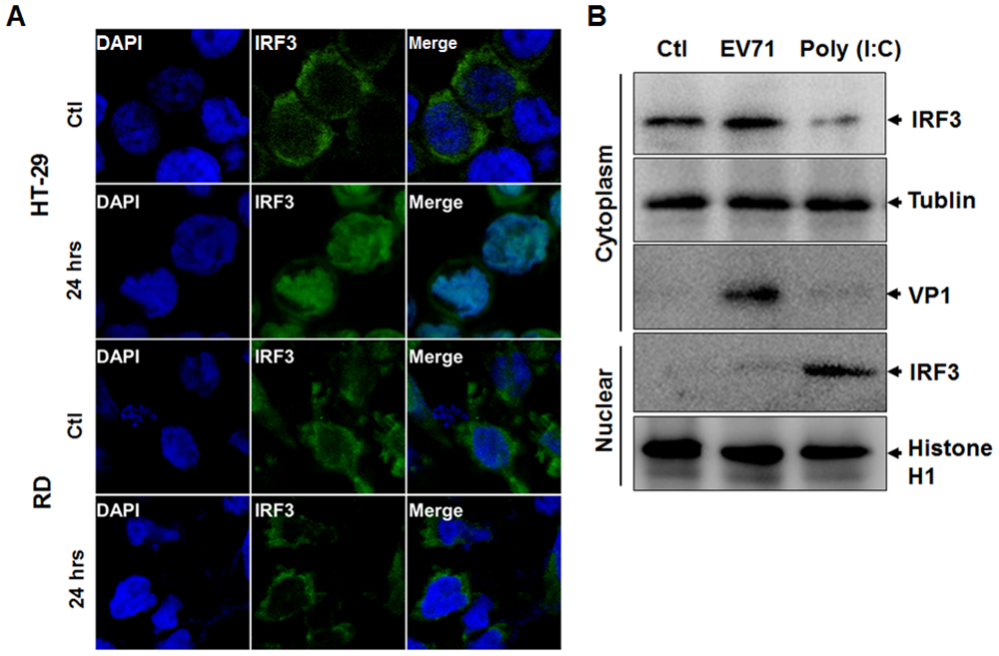
Activation and translocation of IRF3 in HT-29 but not in RD cells. **A.** HT-29 cells and RD cells infected with or without EV71 and the cells were fixed at 24 hrs p.i. The fixed cells were incubated with anti-EV71 VP1 and anti-IRF3 antibodies, followed by staining with conjugated secondary antibodies and DAPI, before being subjected to confocal laser scanning microscopy. **B**. RD cells were infected with EV71 for 24 hrs or stimulated with 50 μg poly (I:C) /ml for 4 hrs at 37°C. Unstimulated and uninfected cells were used as controls. Cell lysates and nuclear extracts were prepared and subjected to SDS-PAGE and western blots assays with specific antibodies.

Differential activation of IRF3 between HT-29 and RD cells was also observed by confocal microscopy. HT-29 and RD cells were infected with EV71, fixed at 24 hrs p.i. and stained with anti-IRF3 antibodies. As shown in Fig 3A, IRF3 was translocated into the nucleus in infected HT-29 cells, compared with the uninfected control. However, in EV71-infected RD cells, IRF3 remained in the cytoplasm (Fig 3A). IRF3 appeared to be also upregulated as shown in its intensity in HT-29 cells infected with EV71, the consequence of the IFN response, but the levels of IRF3 did not change in RD cells.

We also analyzed the distribution of IRF3 in cytoplasmic and nuclear fractions, respectively, in EV71-infected or poly (I:C)-stimulated RD cells. As shown in Fig 3B, when RD cells were stimulated with poly (I:C), IRF3 was translocated into the nuclear faction while its presence in the cytoplasm reduced; however, in EV71-infected cells indicated by the staining with the anti-VP1 antibody, little IRF3 was detected in the nuclear fraction, which remained unchanged in the cytoplasm, indicating that IRF3, retained in the cytoplasm, was blocked from translocation into the nucleus during EV71 infection (Fig 3B).

### Degradation of MAVS in both HT-29 and RD cells infected with EV71

**Fig 4:**
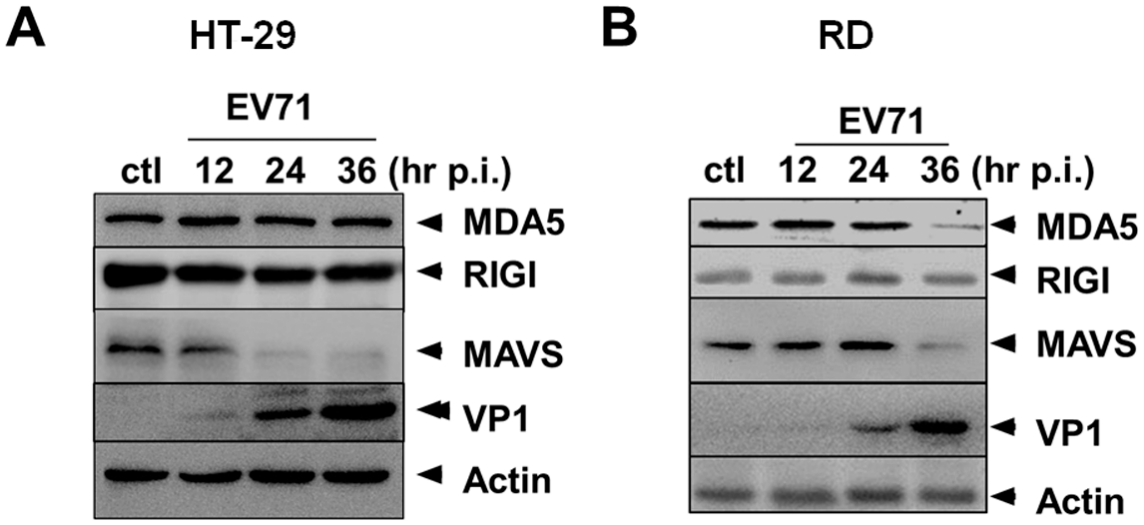
Regulation of RLR signaling in HT-29 and RD cells. HT-29 **(A)** and RD cells **(B)** were infected with EV71 and the cell lysates were prepared at indicated time points, and subsequently subjected to SDS-PAGE and western blot analysis for detecting levels of proteins in RLR signaling pathway.

To examine whether the induction of IFNs is mediated through RLR signaling pathway in HT-29 or RD cells, we infected cells with EV71 at an m.o.i. of 2, and cell lysates were prepared at 12, 24, and 36 hrs p.i. for western blot analysis with specific antibodies for MDA5, RIG-I, and MAVS. As shown in Fig 4, the protein level of MDA5 remained unchanged in HT-29, but decreased at the late stage of infection in RD cells, while RIG-I was not altered, indicating that the receptors were not affected by degradation mediated by viral proteases. The downstream MAVS also decreased at the late stage of infection in RD, where it started to decrease at 12 hrs and almost became undetectable after 24 hrs p.i. in EV71-infected HT-29 cells. Considering IFNs were significantly induced in response to EV71 infection and the degradation of MAVS, the induction is most likely not mediated through RLR signaling, since MAVS was severely degraded in HT-29 cells. In RD cells, although MAVS did not degrade as strongly as in HT-29, IFN induction was deficient, indicating that both RLR as well as TLR signaling failed.

### TRIF is essential to the antiviral response in EV71-infected HT-29 cells

**Fig 5:**
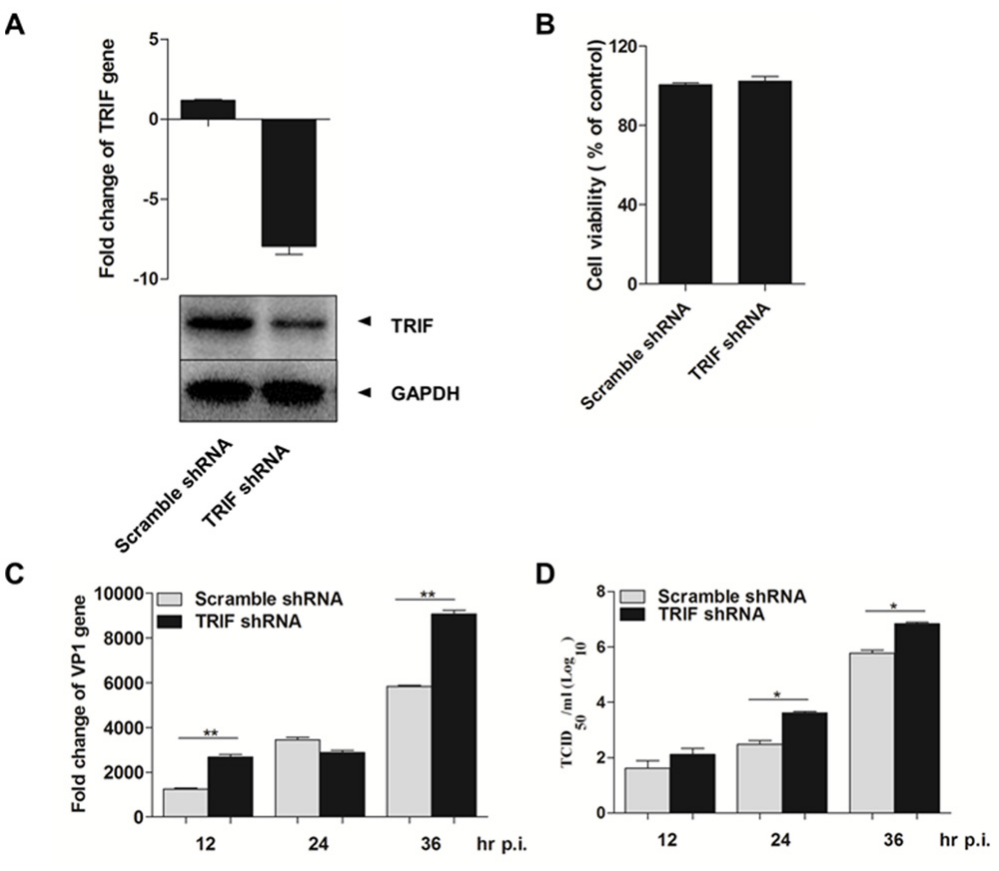
Silence of TRIF facilitated EV71 infection in HT-29 cells. **A**. Knockdown of TRIF in HT-29 cells. Total RNA was extracted from HT-29-CTL and HT-29-TRIF cells. TRIF mRNA transcripts were measured by realtime RT-PCR with primers for TRIF (top). Cell lysates from HT-29-CTL and HT-29-TRIF were prepared for SDS-PAGE and western blot analysis with anti-TRIF antibody (bottom). **B**. Cell viability of lentivirus-infected HT-29 cells as measured by the MTT assay. **C**. Increased viral VP1 gene expression in TRIF-silenced HT-29. HT-29-CTL and HT-29-TRIF cells were infected with EV71 with an m.o.i. of 2, and total RNA were prepared at 12, 24, and 36 hrs p.i. for RT-PCR with specific primers for viral VP1 gene. **D**. EV71 viral replication decreased in TRIF-silenced HT-29 cells. HT-29-CTL and HT-29-TRIF cells were infected with EV71 and the supernatants were collected at 12, 24, and 36 hrs for infectious viral titration at Vero cells in a standard TCID_50_ assay.

To further characterize the essential role of TLR-TRIF-TBK1 signaling in the induction of IFNs in HT-29 cells, we used a lentiviral vector-mediated shRNA to knockdown TRIF expression. The lentiviral vector plasmid pGIPZ-shRNA, encoding the shRNA specific for TRIF gene, was used to generate infectious viruses for infection of HT-29 cells, resulting in cells with reduced TRIF expression in both gene transcripts and protein levels (Fig 5A). Cellular viability remained unchanged, evaluated by an MTT assay, in silenced cells (Fig 5B).

We infected HT-29 cells with EV71 at an m.o.i. of 2, and at 12, 24, and 36 hrs p.i. collected culture medium before preparing total RNA from infected cells. Total RNA was used for measurement of viral VP1 gene copy numbers by realtime PCR, which showed significantly higher VP1 gene expression in silenced HT-29 cells (Fig 5C). We also titrated infectious virus titers in the culture medium, and as shown in Fig 5D, significantly higher titers of infectious viruses were detected at 24 and 36 hrs p.i. in silenced cells, indicating that TRIF is critical to the induction of antiviral immunity to suppress the EV71 replication in infected HT-29 cells.

**Fig 6:**
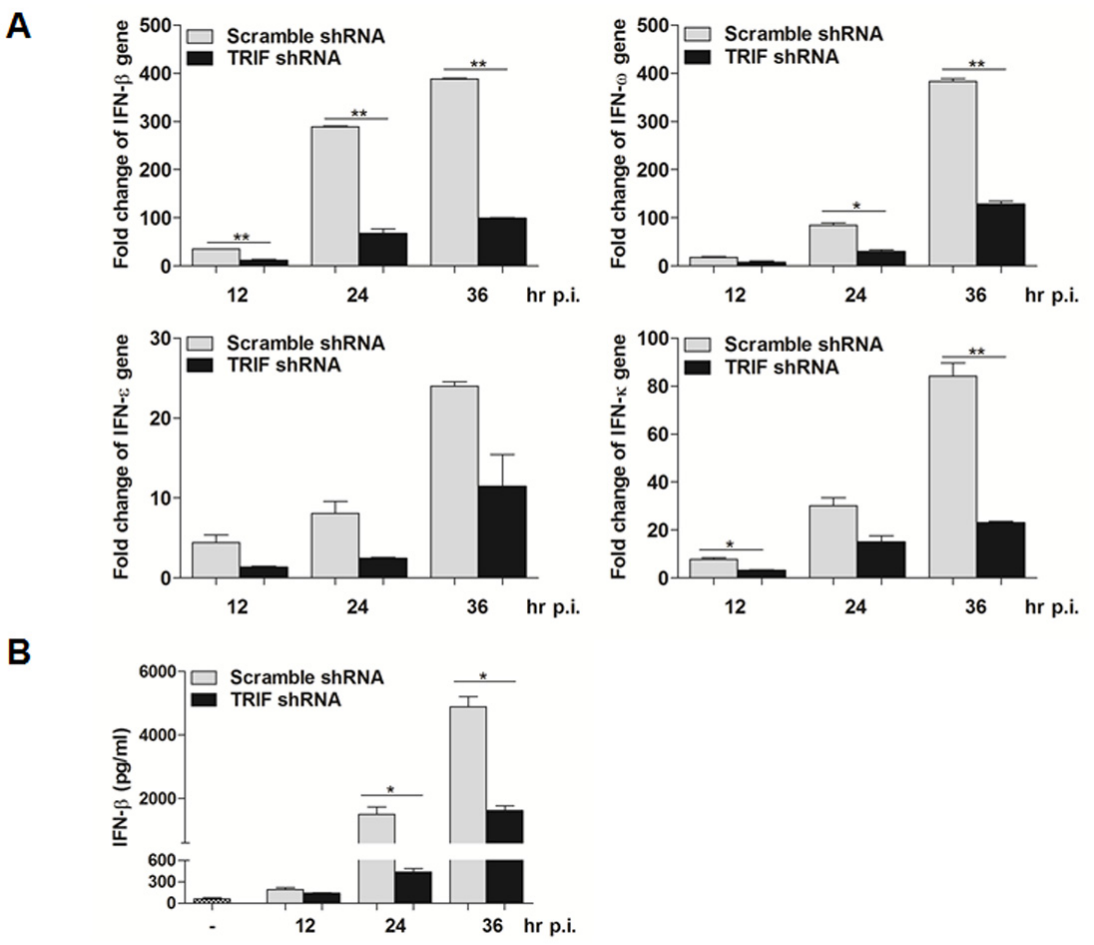
Silence of TRIF reduced the induction of IFNs in EV71-infected HT-29 cells. **A**. HT-29-CTL and HT-29-TRIF cells were infected with EV71 with an m.o.i. of 2, and total RNA were prepared 12, 24, and 36 hrs p.i. for realtime RT-PCR with primers specific for various IFNs (IFN-α, β, ω, ε, κ, λ1, λ2, and λ3). **B** Cultural supernatants were collected for measuring protein levels of IFN-β by ELISA assay.

We further evaluated the role of TRIF in IFN production in EV71-infected HT-29 cells. Total RNA from infected cells or non-infected cells was prepared at 12, 24, and 36 hrs p.i. for realtime RT-PCR to measure transcripts of IFN genes with specific primers for IFN-β, -κ, -ω, and -ε. Induction of IFN-β, -κ, -ω, and -ε, was significantly suppressed in TRIF silenced cells (Fig 6A). We also collected culture medium from infected cells at various times for detection of IFN-β by ELISA. Our data showed that IFN-β decreased significantly, especially at 24 and 36 hrs p.i. (Fig 6B), demonstrating that TRIF is essential to antiviral IFN induction in EV71-infected HT-29 cells.

**Fig 7:**
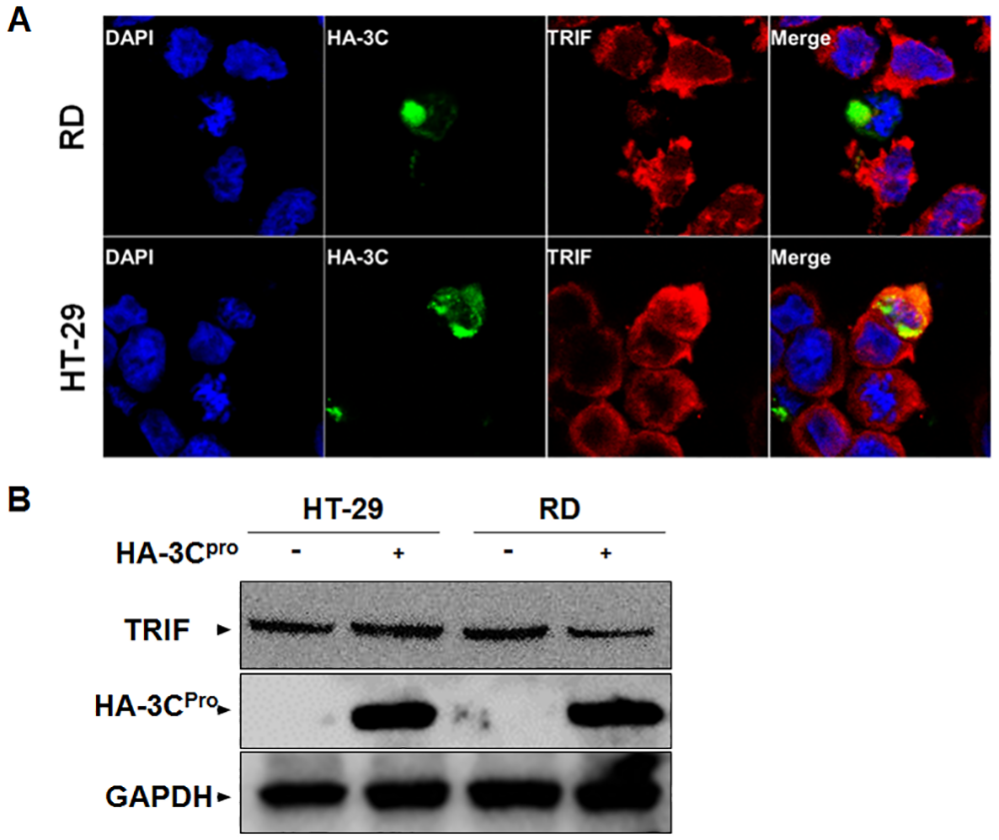
Degradation of TRIF by viral 3C^pro^ in RD but not in HT-29 cells. **A.** RD and HT-29 cells were transfected with a plasmid expressing HA-3C for 24 hrs, and then the cells were fixed and incubated with anti-HA and anti-TRIF antibodies, which were followed by staining with conjugated secondary antibodies and DAPI, before being subjected to laser scanning microscopy in an Olympus confocal microscope. **B**. RD cells and HT-29 cells were transfected with a plasmid expressing HA-3C for 36 hrs. Cell lysates were collected and used for western blot analysis to determine the protein levels of TRIF and 3C^pro^. GAPDH was detected as a loading control.

In EV71 infection, viral proteases such as 3C^pro^ and 2A^pro^ have been shown to be capable of cleaving and degrading host signaling proteins. It has been reported that in HeLa and RD cells, EV71 3C^pro^ cleaved TRIF, resulting in the suppression of TBK1 activation [18]. We examined whether TRIF might also be the target for viral 3C^pro^ in HT-29 cells. We transfected HT-29 and RD cells with an expression plasmid encoding an HA-tagged 3C cDNA for 24 hrs, and then fixed the cells for staining with anti-TRIF antibodies for confocal immunofluorescence microscopy. As shown in Fig 7A, in the 3C^pro^-expressing RD cell the level of cellular TRIF was almost undetectable (top panels, arrow). However, in the 3C^pro^ expressing HT-29 cells, the level of TRIF was comparable to that of the neighboring cells without 3C^pro^ expression (bottom panels, arrow). We also examined the levels of TRIF in HT-29 and RD cells, respectively, with or without the presence of 3C^pro^ by western blot analysis. As shown in Fig 7B, the level of TRIF decreased significantly in RD cells with 3C^pro^, in comparison to the cells without 3C^pro^. However, the levels of TRIF remained unchanged in HT-29 cells, with or without 3C^pro^. In addition, 3C^pro^ appeared not to be co-localized with TRIF although both were present in the cytoplasm as shown in Fig 7A (bottom panels). Taken together, our data indicate that TRIF was degraded by viral 3C^pro^ in RD cells, but was not cleaved or degraded in HT-29 cells. Thus TRIF, which may be uniquely present in human HT-29 cells, might play a critical role in the host innate response during EV71 infection.

## Discussion

Following the eradication of poliovirus, EV71 has emerged as an important neurotropic virus clinically, and no effective antiviral drugs are currently available. Although EV71 is shown to replicate initially in the gastrointestinal tract before spreading to muscle and the central nervous system [27], few clinical intestinal symptoms or signs are observed in patients. Host responses, especially the antiviral response induced in EV71 infection in the intestinal epithelium, may play a critical role in viral pathogenesis and clinical prognosis. We have previously reported that, unlike in RD or HeLa cells in which antiviral IFN response was effectively suppressed, robust IFN-β was induced in EV71-infected human intestinal epithelial cells [19]. Here, we showed that not only the IFN-β, but also the other type I IFNs, including IFN-α, -ε, -κ, and -ε, and type III IFNs, including IFN-λ1, -λ2, and -λ3, were remarkably upregulated and induced to high levels in HT-29 cells compared to those in HeLa and RD cells (Fig 1). A full spectrum of IFNs including all from type I, II, and III, could be induced in response to EV71 in the intestinal epithelial cells, which may be significant in viral pathogenesis and relevant to disease course, clinical development, and prognosis. Only in patients with a deficient immune response would a systemic infection leading to the nervous system occur. In this study we sought to elucidate the mechanism by which host innate immunity remains intact in the intestinal epithelium in response to EV71 infection.

We compared the signaling pathways that mediate IFN response to EV71 infection in HT-29 and RD cells and found that TRIF, the key signaling player in TLR signaling, was not cleaved by viral 3C^pro^ in HT-29, unlike in RD cells where EV71 infection induced significant degradation of TRIF. Our data showed that TLR appeared to be the essential signaling pathway for IFN response, since MAVS was drastically degraded, which may have crippled the RLR signaling. Indeed, in TRIF-silenced HT-29 cells, replication of EV71 increased significantly in parallel to the decrease of all IFNs. Thus, a TLR3-TRIF axis appears to be essential and responsible for robust induction of IFNs in HT-29 cells.

Type I IFNs serve as first line defense in innate immunity, implicated for the induction of hundreds of interferon-stimulated genes (ISGs) that play a key role in host resistance to viral infections. Induction of IFNs or other cytokines also facilitates adaptive immune responses for the host to eventually clear the viruses. Previous studies showed that type I IFNs contribute to protection against EV71 infection by controlling viral replication [28], but in RD or HeLa cells, little IFNs were produced during EV71 infection. Efforts have been made to explore the mechanism by which EV71 suppresses cellular responses, and have found viral-encoded proteases to be involved. In HeLa and RD cells, 3C^pro^ of EV71 can bind to RIG-I, thus disrupting its interaction with MAVS and blocking the RLR signaling from activation and nuclear translocation of IRF3 [20]. EV71 3C^pro^, as a protease, was also found to be able to cleave and degrade TRIF and IRF7, and therefore effectively impair type I IFN response through TLR signaling in these cells [18, 29]. Viral 2A^pro^, another protease encoded by EV71, is capable of targeting and degrading MAVS, impeding RLR signaling as well for IFN induction in EV71-infected HeLa cells [30], in which 2A^pro^ also enhances MDA5 degradation, further suppressing the activation of IRF3 [31].

The IFN response is mainly regulated by RLR or TLR signaling pathways. The RLR pathway is effectively suppressed not only in HeLa or RD cells, but also in HT-29 cells. Differential regulation of TLR signaling in HeLa, RD, and HT-29 cells is of particular interest in indicating the importance of cell or tissue types that may be implicated in disease progress clinically. Severe cases of EV71 infection might be due to impaired TLR signaling in the gut, which would therefore impede the critical defense and result in the virus evasion and then spread systemically.

TLR signaling can be MyD88-dependent (TLR7/8) or MyD88-independent (TLR3), using the adaptor proteins MyD88 and TRIF, respectively. The ligands for TLR7/8 are single-stranded RNA [32], while the ligands for TLR3 are dsRNA, which could be viral intermediate replicative RNA produced during viral RNA replication. In TLR signaling the adaptor protein for TLR7/8 is MyD88, which interacts with TLR7, while the adaptor protein for TLR3 is TRIF, which activates TRAF3 and TBK1. TRIF was significantly degraded as observed beginning at 12 hrs and was barely detectable at 36 hrs p.i. in EV71-infected RD cells, but the levels of TRIF remained largely consistent in HT-29 cells (Fig 2). IRF7 was also remarkably degraded at 24 hrs and almost undetectable at 36 hrs in RD cells, suggesting that TLR signaling is completely blocked, through either MyD88-dependent or MyD88-independent in RD cells. However, in HT-29 cells the levels of IRF7 decreased marginally at 36 hrs, but were shown unchanged at 24 hrs p.i. (Fig 2), suggesting that TLR signaling may remain intact and be unaffected, and that TLR7/8 signaling may also function early and decrease only at the late stage of infection in HT-29 cells. We cannot determine which TLRs would be involved or more important, since we have not identified whether TLR3 or TLR7/8 responds to EV71 infection.

Interestingly, TLR9, discerning unmethylated CpG sequences and pathogen-derived DNA, was recently reported to mediate signaling for induction of IFNs and protection in EV71-infected mice by recognizing danger-associated molecular patterns released during infection [33]. TLR9 is mediated through a MyD88-dependent pathway and TLR7, as for TLR7/8, and thus TLR9-mediated signaling remains largely functional. We have also data indicating that MyD88 levels were unaffected in EV71-infected HT-29 cells (data not shown).

TLR signaling is mediated by TRAF3 or TRAF6, which can subsequently activate TBK1/IKKε and IRF3 for IFN induction, or NF-κB and MAP kinase via TAK1 [34]. Both TRAF3 and TRAF6 are largely unchanged in their expression levels in EV71-infected HT-29 cells (Fig 2). TLR signaling through either MyD88-independent via TRIF or MyD88-dependent via TRAF3 pathways can both activate TBK1/IKKε, leading to IRF3 phosphorylation and translocation into the nucleus in EV71-infected HT-29 cells, which did not occur in RD cells (Fig 3). TLR signaling through a MyD88-dependent via TRAF6 pathway activated TAK1, leading to activation of NFκB and MAP kinase signaling pathways, which was also reported in our previous studies [23]. We showed that NF-κB signaling was activated in EV71 infection, which led to increased viral replication and proinflammatory cytokine induction, while the induction of IFN-β was positively regulated by both ERK1/2 and JNK1/2 [23]. Our current study further demonstrates that TLR-TRAF6 signaling is functional when TAK1 is phosphorylated and activated (Fig 2C) and most likely involved in the activation of both NF-κB and MAP kinases for innate immune responses in HT-29 cells. However, TLR-TRAF6 signaling may have been severely suppressed, because the levels of TAK1 were lower after 24 hrs p.i., and more dramatically, the TAK1 phosphorylation or activation was completely inhibited in EV71-infected RD cells (Fig 2D).

We also examined the expression levels of TLR3 before and after the infection in RD and HT-29 cells. While the expression of TLR3 remained unchanged in RD cells, we observed an increase of the TLR3 transcripts up to 2 to 6 fold in HT-29 cells within 36 hrs post EV71 infection (data not shown). The increase of TLR3 in HT-29 cells may be due to the induced IFN in HT-29 cells. It is difficult, however, to estimate the impact of the increased TLR3 on the further induction of IFN since the basal level of TLR3 may have been sufficient enough for EV71-stimulated TLR3 signaling in HT-29 cells.

Can the robust IFN response in EV71-infected HT-29 cells be attributed to the resistance of TRIF to 3C degradation without other components involved in the differential IFN signaling? Almost no differences were observed in the levels of RIG-1 and MDA5 in both HT-29 and RD cells infected with EV71 (Fig 4A and 4B). MAVS was effectively degraded in infected HT-29 cells (Fig 4A), indicates that RIG-I/MDA5 signaling may not be essential to IFN induction in HT-29 cells, although MAVS was more resistant to degradation in infected RD cells. There was no difference in the level of TRAF3, which is responsible for IRF3 activation between HT-29 and RD cells infected with EV71 (Fig 2A and 2B). The Levels of TRAF6 remains unchanged in HT-29 cells but degraded in the late stage of infection only in RD cells (Fig 2B). However, TRAF6 is mainly responsible for activation of TAK1 and NFκB signaling, which plays a minor role in IFN induction. The key difference is the level of TRIF, which was significantly degraded starting in RD cells (Fig 2B). In addition, Functional knockdown of TRIF confirmed the role of TRIF in IFN induction in HT-29 (Figs 5 and 6); and viral 3C degraded TRIF in RD cells but failed in HT-29 cells, transfected with plasmids expressing 3C (Fig 7B). We hypothesize that viral 3C interacts with a host protein in HT-29 or intestinal epithelial cells and therefore, the function of viral 3C is blocked and its degradation of TRIF is sabotaged in EV71 infection. Differential regulation of host responses in different cell or tissue types may play a key role in EV71 viral pathogenesis and be related to manifestation and patient prognosis clinically.

## Supporting Information

S1 Fig Semi-quantitative analysis of TRIF, TRAF6 and IRF7 expression in HT-29 and RD cells infected with EV71. HT-29 and RD cells were infected with EV71 and the cell lysates were prepared for western blot analyses as described in Fig 2. Gray scanning of the proteins bands in blots and relative expression levels of TRIF (**S1A**), TRAF6 (**S1B**), and IRF7 (**S1C**) against actin controls at each time points, respectively, were shown.
(PDF)

## References

[1] Solomon T, Lewthwaite P, Perera D, Cardosa MJ, McMinn P, Ooi MH. Virology, epidemiology, pathogenesis, and control of enterovirus 71. The Lancet infectious diseases. 2010;10(11):778–90. 10.1016/S1473-3099(10)70194-8 20961813

[2] Lum LC, Wong KT, Lam SK, Chua KB, Goh AY, Lim WL, et al Fatal enterovirus 71 encephalomyelitis. J Pediatr. 1998;133(6):795–8. Epub 1998/12/08. . 9842048 10.1016/s0022-3476(98)70155-6

[3] McMinn PC. An overview of the evolution of enterovirus 71 and its clinical and public health significance. FEMS microbiology reviews. 2002;26(1):91–107. Epub 2002/05/15. . 12007645 10.1111/j.1574-6976.2002.tb00601.x

[4] Yi L, Lu J, Kung H-f, He M-L. The virology and developments toward control of human enterovirus 71. Critical reviews in microbiology. 2011;37(4):313–27. 10.3109/1040841X.2011.580723 21651436

[5] Takaoka A, Yanai H. Interferon signalling network in innate defence. Cellular microbiology. 2006;8(6):907–22. Epub 2006/05/10. 10.1111/j.1462-5822.2006.00716.x . 16681834

[6] Sheppard P, Kindsvogel W, Xu W, Henderson K, Schlutsmeyer S, Whitmore TE, et al IL-28, IL-29 and their class II cytokine receptor IL-28R. Nature immunology. 2003;4(1):63–8. Epub 2002/12/07. 10.1038/ni873 . 12469119

[7] Kawai T, Akira S. TLR signaling. Cell death and differentiation. 2006;13(5):816–25. Epub 2006/01/18. 10.1038/sj.cdd.4401850 . 16410796

[8] Rothenfusser S, Goutagny N, DiPerna G, Gong M, Monks BG, Schoenemeyer A, et al The RNA helicase Lgp2 inhibits TLR-independent sensing of viral replication by retinoic acid-inducible gene-I. J Immunol. 2005;175(8):5260–8. Epub 2005/10/08. . 16210631 10.4049/jimmunol.175.8.5260

[9] Yoneyama M, Kikuchi M, Matsumoto K, Imaizumi T, Miyagishi M, Taira K, et al Shared and unique functions of the DExD/H-box helicases RIG-I, MDA5, and LGP2 in antiviral innate immunity. J Immunol. 2005;175(5):2851–8. Epub 2005/08/24. . 16116171 10.4049/jimmunol.175.5.2851

[10] Tatematsu M, Seya T, Matsumoto M. Beyond dsRNA: Toll-like receptor 3 signalling in RNA-induced immune responses. The Biochemical journal. 2014;458(2):195–201. Epub 2014/02/15. 10.1042/BJ20131492 . 24524192

[11] Thompson MR, Kaminski JJ, Kurt-Jones EA, Fitzgerald KA. Pattern recognition receptors and the innate immune response to viral infection. Viruses. 2011;3(6):920–40. Epub 2011/10/14. 10.3390/v3060920 21994762 PMC3186011

[12] Sato S, Sugiyama M, Yamamoto M, Watanabe Y, Kawai T, Takeda K, et al Toll/IL-1 receptor domain-containing adaptor inducing IFN-beta (TRIF) associates with TNF receptor-associated factor 6 and TANK-binding kinase 1, and activates two distinct transcription factors, NF-kappa B and IFN-regulatory factor-3, in the Toll-like receptor signaling. J Immunol. 2003;171(8):4304–10. Epub 2003/10/08. . 14530355 10.4049/jimmunol.171.8.4304

[13] Meylan E, Burns K, Hofmann K, Blancheteau V, Martinon F, Kelliher M, et al RIP1 is an essential mediator of Toll-like receptor 3-induced NF-kappa B activation. Nature immunology. 2004;5(5):503–7. Epub 2004/04/06. 10.1038/ni1061 . 15064760

[14] Hacker H, Redecke V, Blagoev B, Kratchmarova I, Hsu LC, Wang GG, et al Specificity in Toll-like receptor signalling through distinct effector functions of TRAF3 and TRAF6. Nature. 2006;439(7073):204–7. Epub 2005/11/25. 10.1038/nature04369 . 16306937

[15] Akira S, Uematsu S, Takeuchi O. Pathogen recognition and innate immunity. Cell. 2006;124(4):783–801. Epub 2006/02/25. 10.1016/j.cell.2006.02.015 . 16497588

[16] Akira S, Takeda K. Toll-like receptor signalling. Nature reviews Immunology. 2004;4(7):499–511. Epub 2004/07/02. 10.1038/nri1391 . 15229469

[17] O'Neill LA, Bowie AG. The family of five: TIR-domain-containing adaptors in Toll-like receptor signalling. Nature reviews Immunology. 2007;7(5):353–64. Epub 2007/04/26. 10.1038/nri2079 . 17457343

[18] Lei X, Sun Z, Liu X, Jin Q, He B, Wang J. Cleavage of the adaptor protein TRIF by enterovirus 71 3C inhibits antiviral responses mediated by Toll-like receptor 3. Journal of virology. 2011;85(17):8811–8. 10.1128/JVI.00447-11 21697485 PMC3165803

[19] Chi C, Sun Q, Wang S, Zhang Z, Li X, Cardona CJ, et al Robust Antiviral Responses to Enterovirus 71 Infection in Human Intestinal Epithelial Cells. Virus research. 2013. 10.1016/j.virusres.2013.05.002 23685430

[20] Lei X, Liu X, Ma Y, Sun Z, Yang Y, Jin Q, et al The 3C protein of enterovirus 71 inhibits retinoid acid-inducible gene I-mediated interferon regulatory factor 3 activation and type I interferon responses. Journal of virology. 2010;84(16):8051–61. 10.1128/JVI.02491-09 20519382 PMC2916543

[21] Kärber G. Beitrag zur kollektiven Behandlung pharmakologischer Reihenversuche. Naunyn-Schmiedebergs Archiv für experimentelle pathologie und pharmakologie. 1931;162(4):480–3.

[22] Livak KJ, Schmittgen TD. Analysis of relative gene expression data using real-time quantitative PCR and the 2(-Delta Delta C(T)) Method. Methods. 2001;25(4):402–8. Epub 2002/02/16. [pii]. . 11846609 10.1006/meth.2001.1262

[23] Wang C, Gao L, Jin Y, Cardona CJ, Xing Z. Regulation of host responses and viral replication by the mitogen-activated protein kinases in intestinal epithelial cells infected with Enterovirus 71. Virus Res. 2015;197:75–84. Epub 2014/12/23. 10.1016/j.virusres.2014.12.016 . 25529441

[24] Gurtler C, Bowie AG. Innate immune detection of microbial nucleic acids. Trends in microbiology. 2013;21(8):413–20. Epub 2013/06/04. 10.1016/j.tim.2013.04.004 23726320 PMC3735846

[25] Takaoka A, Taniguchi T. New aspects of IFN-alpha/beta signalling in immunity, oncogenesis and bone metabolism. Cancer science. 2003;94(5):405–11. Epub 2003/06/26. . 12824884 10.1111/j.1349-7006.2003.tb01455.x PMC11160234

[26] Chattopadhyay S, Sen GC. Tyrosine phosphorylation in Toll-like receptor signaling. Cytokine & growth factor reviews. 2014;25(5):533–41. Epub 2014/07/16. 10.1016/j.cytogfr.2014.06.002 25022196 PMC4254339

[27] Chen CS, Yao YC, Lin SC, Lee YP, Wang YF, Wang JR, et al Retrograde axonal transport: a major transmission route of enterovirus 71 in mice. Journal of virology. 2007;81(17):8996–9003. 17567704 10.1128/JVI.00236-07 PMC1951457

[28] Liu ML, Lee YP, Wang YF, Lei HY, Liu CC, Wang SM, et al Type I interferons protect mice against enterovirus 71 infection. The Journal of general virology. 2005;86(Pt 12):3263–9. Epub 2005/11/22. 10.1099/vir.0.81195-0 . 16298971

[29] Lei X, Xiao X, Xue Q, Jin Q, He B, Wang J. Cleavage of interferon regulatory factor 7 by enterovirus 71 3C suppresses cellular responses. J Virol. 2013;87(3):1690–8. Epub 2012/11/24. 10.1128/JVI.01855-12 23175366 PMC3554134

[30] Wang B, Xi X, Lei X, Zhang X, Cui S, Wang J, et al Enterovirus 71 protease 2Apro targets MAVS to inhibit anti-viral type I interferon responses. PLoS pathogens. 2013;9(3):e1003231 Epub 2013/04/05. 10.1371/journal.ppat.1003231 23555247 PMC3605153

[31] Kuo RL, Kao LT, Lin SJ, Wang RY, Shih SR. MDA5 plays a crucial role in enterovirus 71 RNA-mediated IRF3 activation. PloS one. 2013;8(5):e63431 Epub 2013/05/08. 10.1371/journal.pone.0063431 23650567 PMC3641126

[32] Uematsu S, Sato S, Yamamoto M, Hirotani T, Kato H, Takeshita F, et al Interleukin-1 receptor-associated kinase-1 plays an essential role for Toll-like receptor (TLR)7- and TLR9-mediated interferon-{alpha} induction. J Exp Med. 2005;201(6):915–23. Epub 2005/03/16. 10.1084/jem.20042372 15767370 PMC2213113

[33] Hsiao HB, Chou AH, Lin SI, Chen IH, Lien SP, Liu CC, et al Toll-like receptor 9-mediated protection of enterovirus 71 infection in mice is due to the release of danger-associated molecular patterns. J Virol. 2014;88(20):11658–70. Epub 2014/08/01. 10.1128/JVI.00867-14 25078697 PMC4178751

[34] Lester SN, Li K. Toll-like receptors in antiviral innate immunity. J Mol Biol. 2014;426(6):1246–64. Epub 2013/12/10. 10.1016/j.jmb.2013.11.024 24316048 PMC3943763

