# Supplementary material for: Differential Regulation of TLR Signaling on the Induction of Antiviral Interferons in Human Intestinal Epithelial Cells Infected with Enterovirus 71
Source: PLoS One. 2016 Mar 23;11(3):e0152177. doi: 10.1371/journal.pone.0152177 (PMC4805281; doi:10.1371/journal.pone.0152177)

S1 Figure Semi-quantitative analysis of TRIF, TRAF6 and IRF7 expression in HT-29 and RD cells infected with EV71.

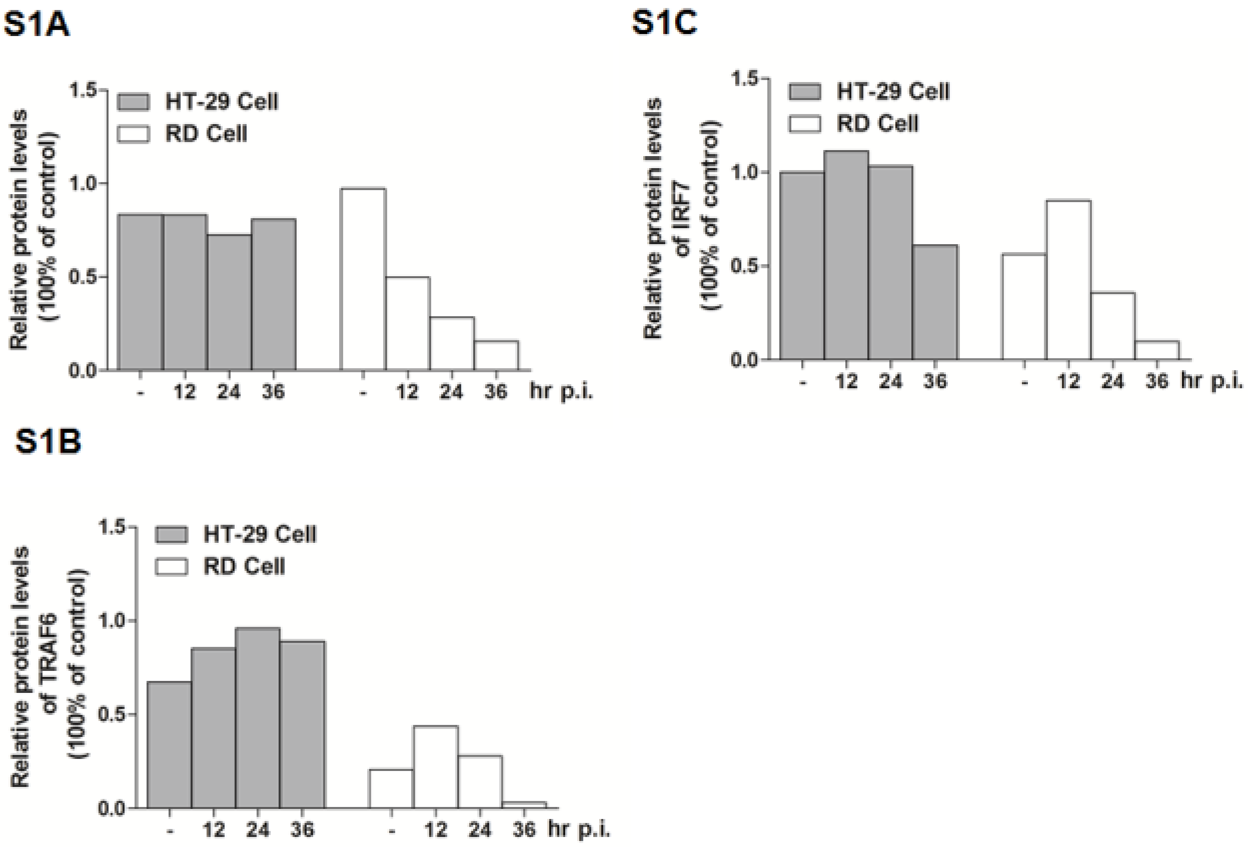

Supplement: S1 Fig — HT-29 and RD cells were infected with EV71 and the cell lysates were prepared for western blot analyses as described in Fig 2. Gray scanning of the proteins bands in blots and relative expression levels of TRIF (S1A), TRAF6 (S1B), and IRF7 (S1C) against actin controls at each time points, respectively, were shown. (PDF) [file pone.0152177.s001.pdf]
